# HMGB1 is a mediator of cuproptosis-related sterile inflammation

**DOI:** 10.3389/fcell.2022.996307

**Published:** 2022-09-21

**Authors:** Jiao Liu, Yang Liu, Yuan Wang, Rui Kang, Daolin Tang

**Affiliations:** ^1^ DAMP Laboratory, Third Affiliated Hospital of Guangzhou Medical University, Guangzhou, Guangdong, China; ^2^ Department of Surgery, UT Southwestern Medical Center, Dallas, TX, United States

**Keywords:** cuproptosis, HMGB1, DAMP, ager, AMPK, inflammation

## Abstract

Cuproptosis is a recently recognized modality of cell death driven by intracellular copper-dependent mitochondrial stress. However, the mediators of the sterile inflammatory response to cuproptotic death are undetermined. Here, we report that high-mobility group box 1 (HMGB1), a damage-associated molecular pattern, is released by cuproptotic cells to initiate inflammation. Mechanically, copper accumulation-induced adenosine triphosphate (ATP) depletion activates AMP-activated protein kinase (AMPK) to promote HMGB1 phosphorylation, resulting in increased extracellular release. In contrast, genetic (using RNAi) or pharmacologic (using dorsomorphin) inhibition of AMPK activation limits cuproptosis and HMGB1 release. Functionally, the ability of HMGB1-deficient cuproptotic cells to promote advanced glycosylation end product-specific receptor (AGER, also known as RAGE)-dependent inflammatory cytokine production is greatly reduced. Thus, HMGB1 is a key immune mediator of cuproptosis-initiated sterile inflammation.

## Introduction

Cell death is not only a physiological process during organ development, but also a pathological event associated with tissue damage and sterile inflammation (Rock and Kono, 2008). Although historically there have been many classifications for cell death, it is now divided into accidental cell death and regulated cell death (Tang et al., 2019). Regulated cell death has various forms, exhibiting different triggering signals, biochemical events, and molecular effectors (Tang et al., 2019). Cuproptosis is the newest member of the regulated cell death family, a type of mitochondrial cell death first defined in 2022 that is the result of intracellular copper accumulation (Tsvetkov et al., 2022). The cuproptotic process depends on mitochondrial proteotoxic stress rather than copper-induced mitochondrial oxidative stress (Tsvetkov et al., 2022). Genome-scale CRISPR-Cas9 knockout screening revealed that ferredoxin 1 (FDX1) protein plays a significant role in promoting cuproptosis through mitochondrial protein lipoylation (Tsvetkov et al., 2022). However, the immune consequences of cuproptotic death remain unclear.

High-mobility group box 1 (HMGB1) is the most abundant nonhistone nuclear protein and plays a fundamental role in maintaining nucleosome structure and function, affecting gene transcription, DNA repair, and chromosomal rearrangement (Lotze and Tracey, 2005; Voong et al., 2021). In addition to being located in the nucleus, HMGB1 can also be present in other organelles as well as in the extracellular space to regulate multiple cell processes (Lotze and Tracey, 2005). Specifically, HMGB1 can be secreted or released by stressed, dead, or dying cells (Wang et al., 1999; Scaffidi et al., 2002; Zhu et al., 2021; Chen et al., 2022). Once released, HMGB1 acts as a damage-associated molecular pattern (DAMP) to trigger immune responses by binding multiple pattern recognition receptors, such as toll-like receptor 4 (TLR4) and advanced glycosylation end product-specific receptor (AGER, also known as RAGE) (Tsung et al., 2007; Kang et al., 2014a; Amornsupak et al., 2022; He et al., 2022; Lei et al., 2022). Targeting the release and activity of HMGB1 is an attractive strategy to limit excessive inflammation in diseases associated with microbial infection and tissue damage (Andersson and Tracey, 2011; Kang et al., 2014b; Andersson et al., 2021; Zhu et al., 2021).

In the study described here, we report a previously unknown function of HMGB1 in mediating cuproptosis-related inflammation. We not only elucidate the molecular mechanism of HMGB1 release during cuproptosis, but also assess the effect of this release from cuproptotic cells on macrophage activation. These findings provide new insights into understanding the molecular links between cuproptotic death and innate immunity.

## Methods

### Reagents

The antibodies to ACTB (#3700), PRKAA (#2532), p-PRKAA (#2535), H3 (#4499), ACACA (#4190), p-ACACA (#3661), and phospho-threonine/tyrosine antibody (#9416) were purchased from Cell Signaling Technology. The antibodies to HMGB1 were purchased from NOVUS (#NB100-2322). Elesclomol (#S1052), tetrathiomolybdate (#E1166), necrostatin-1 (#S8037), ferrostatin-1 (#S7243), MitoQ10 (#S8978), dorsomorphin (#S7306), and Z-VAD-FMK (#S7023) were purchased from Selleck Chemicals. CuCl2 (#222011) was purchased from Sigma-Aldrich. Dimethyl sulfoxide (DMSO; #D5879, Sigma-Aldrich) was used to prepare the stock solution of drugs. The final concentration of DMSO in the drug working solution in the cells was <0.01%. DMSO of 0.01% was used as a vehicle control in cell culture assays.

### Cell culture

Human Calu1 and MIA PaCa2 cell lines were obtained from the American Type Culture Collection. Wild-type (WT) and hmgb1^−/−^ mouse embryonic fibroblasts (MEFs) were kind gifts from Dr. Marco Bianchi. Bone marrow-derived macrophages (BMDMs) were obtained from *ager* ^*−/−*^ (a kind gift from Dr. Angelica Bierhaus) and *tlr4* ^*−/−*^ (#029015, The Jackson Laboratory) mice. Human peripheral blood mononuclear cells (PBMCs) were isolated from blood samples using Ficoll density gradient centrifugation. These cells were grown in Dulbecco’s modified Eagle’s medium or RPMI-1640 medium with 10% fetal bovine serum, 2 mM L-glutamine, and 100 U/ml of penicillin and streptomycin. All cells were mycoplasma-free and authenticated using short tandem repeat DNA profiling analysis.

### RNAi

Predesigned human PRKAA1-shRNA (#TRCN0000000861), PRKAA2-shRNA (#TRCN0000002171), HMGB1-shRNA (#TRCN0000018934), and control empty shRNA (pLKO.1) were obtained from Sigma-Aldrich. RNAi was performed using Lipofectamine 3000 transfection reagent (#L3000015, Thermo Fisher Scientific) according to the manufacturer’s instructions. Puromycin (#A1113803, Thermo Fisher Scientific) selection was used to generate stable cell lines.

### Western blots

Nuclear extraction was performed using a commercial kit (#ab113474, abcam) according to the manufacturer’s protocol. Western blotting was performed as previously described (Tang et al., 2010). In brief, proteins in the whole cell lysate or nuclear extraction were resolved on 12.5% PAGE gels (#PG113, EpiZyme Biotechnology) and transferred to a polyvinylidene difluoride membrane. After blocking with 5% milk at room temperature for 2 h, the membrane was incubated overnight at 4°C with various primary antibodies (1:1000). After incubation with peroxidase-conjugated secondary antibodies (1:1000) for 1 h at room temperature, the signals were visualized using SuperSignal West Pico PLUS Chemiluminescent Substrate (#34580, Thermo Fisher Scientific) and by using a ChemiDoc Touch Imaging System (Bio-Rad).

### Cell death assays

The level of cell death was assayed using a LIVE/DEAD cell viability/cytotoxicity assay kit (#L3224, Thermo Fisher Scientific) according to the manufacturer’s protocol.

### Mitochondrial assay

Mitochondrial membrane potential changes in cells were assessed using membrane-permeant JC-1 dye (#M34152, Thermo Fisher Scientific) according to the manufacturer’s protocol. Briefly, indicated cells were incubated with 2.5 μM JC-1 in a black 96-well plate (#3904, Corning) at 37°C for 15 min (Liu et al., 2022). The fluorescence signals were analyzed on a fluorescent microplate reader (Tecan). The red/green fluorescence ratio was calculated. The untreated group was given a value of 1. Mitochondrial ROS was assayed using MitoSOX probe (#M36008, Thermo Fisher Scientific) according to the manufacturer’s instructions. An ATP Assay Kit (#MAK190, Sigma-Aldrich) was used to quantify the ATP concentration by fluorometric detection of ATP in samples.

### Transmission electron microscopy analysis

Transmission electron microscopy analysis was performed as previously described (Tang et al., 2011; Liu et al., 2022). In brief, cells were fixed with 2% paraformaldehyde and 2% glutaraldehyde in 0.1 mol/L phosphate buffer (pH 7.4), followed by postfixation for 6 h in 1% OsO4. After dehydration with graded alcohols, the sample was embedded in epoxy resin. A thinly cut sample (70 nm) was mounted on copper mesh and post-stained with 2% uranyl acetate and 1% lead citrate, dried, and analyzed with a transmission electron microscope (JEOL).

### qPCR analysis

Total RNA was extracted and purified from cultured cells using the RNeasy Plus Mini Kit (#74136, QIAGEN). First-strand cDNA was synthesized from 1 μg of RNA using the iScript cDNA Synthesis Kit (#1708890, Bio-Rad). Then cDNA from various cell samples were amplified by real-time quantitative polymerase chain reaction (qPCR) with specific primers (mouse *Tnf*: GGT​GCC​TAT​GTC​TCA​GCC​TCT​T and GCC​ATA​GAA​CTG​ATG​AGA​GGG​AG; human *TNF*: CTC​TTC​TGC​CTG​CTG​CAC​TTT​G and ATG​GGC​TAC​AGG​CTT​GTC​ACT​C; mouse *Il6*: TAC​CAC​TTC​ACA​AGT​CGG​AGG​C and CTG​CAA​GTG​CAT​CAT​CGT​TGT​TC; human *IL6*: AGA​CAG​CCA​CTC​ACC​TCT​TCA​G and TTC​TGC​CAG​TGC​CTC​TTT​GCT​G) using a CFX96 Touch Real-Time PCR Detection System (Bio-Rad).

### Immunoprecipitation analysis

Cells were lysed at 4°C in RIPA buffer (#9806, Cell Signaling Technology). Before immunoprecipitation, samples containing equal amounts of proteins were pre-cleared with protein A sepharose and subsequently incubated with various irrelevant IgG or specific anti-HMGB1 antibodies (ab18256, Abcam; 2 μg/ml) in the presence of protein A sepharose beads. The beads were washed three times with RIPA buffer and the immune complexes were eluted from the beads and subjected to SDS–PAGE and immunoblot analysis of phospho-threonine/tyrosine as previously described.

### HMGB1 and LDH analysis

The release of HMGB1 or LDH in cell culture supernatants was assayed using an ELISA kit from Sino-Test Corporation (#ST51011) or Thermo Fisher Scientific (#C20300) according to the manufacturer’s instructions. The phosphorylation of HMGB1 in whole-cell extracts was determined by immunoprecipitation with anti-HMGB1 antibodies, followed by WB assay with anti-phosphothreonine/tyrosine antibodies.

### Statistical analysis

Statistics were calculated with GraphPad Prism 9.01. A *t* test or ANOVA were used for statistical analysis. When the ANOVA was significant, *post hoc* testing of differences between groups was performed using Tukey’s multiple comparisons test. A *p* value of less than 0.05 was considered statistically significant.

## Results

### HMGB1 is released during cuproptosis

**FIGURE 1:**
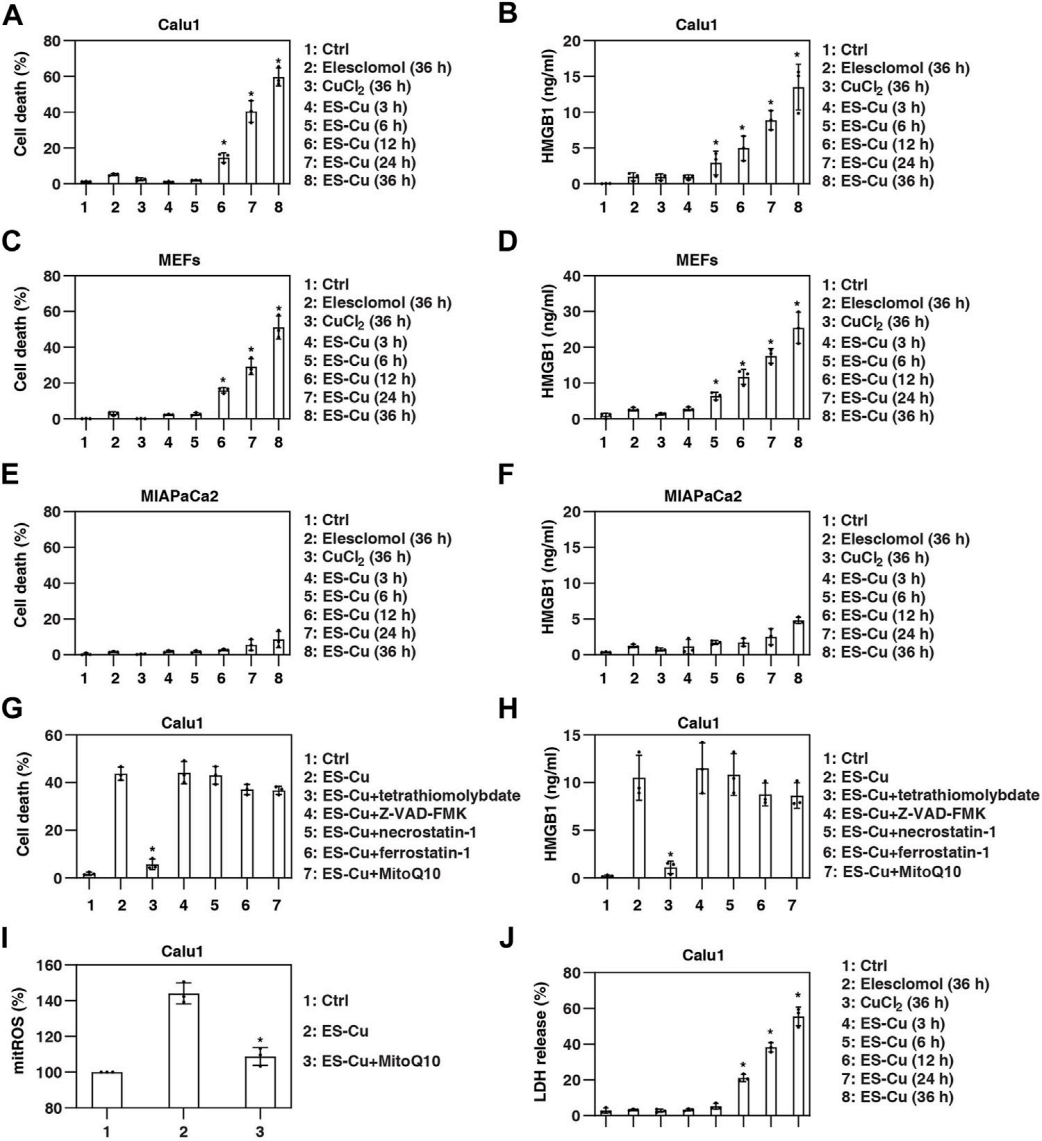
HMGB1 is released during cuproptosis. (A–F) Indicated cells were treated with elesclomol (30 nM), CuCl_2_ (30 nM), or elesclomol + CuCl_2_ (ES-Cu) for 3–36 h and then cell death and HMGB1 release were assayed (*n* = 3; *p < 0.05 versus control group, one-way ANOVA). (G,H) Calu1 cells were treated with ES-Cu (30 nM/30 nM) in the absence or presence of tetrathiomolybdate (20 µM), Z-VAD-FMK (20 µM), necrostatin-1 (20 µM), ferrostatin-1 (1 µM), or MitoQ10 (5 µM) for 24 h. Cell death and HMGB1 release were assayed (*n* = 3; *p < 0.05 versus ES-Cu group, *t* test). (I) Effects of MitoQ10 (5 µM) on mitoROS production in Calu1 cells following treatment with ES-Cu (30 nM/30 nM) for 24 h (*n* = 3; *p < 0.05 versus ES-Cu group, *t* test). (J) Calu1 cells were treated with elesclomol (30 nM), CuCl_2_ (30 nM), or elesclomol + CuCl_2_ (ES-Cu) for 3–36 h and then LDH release were assayed (*n* = 3; *p < 0.05 versus control group, one-way ANOVA). The results in (A–J) are representative of those from three independent experiments.

The classical stimulus for cuproptosis is the combination of the copper-carrier drug elesclomol and CuCl_2_ (Tsvetkov et al., 2022). Consistent with previous studies in lung cancer cells (Tsvetkov et al., 2022), elesclomol and CuCl_2_ alone at 30 nM had no significant effects on the induction of cell death in Calu1 cells (a human lung cancer cell line; Figure 1A). However, the combined use of elesclomol and CuCl_2_ (a combination hereafter referred to as ES-Cu) induced cell death in a time-dependent manner (Figure 1A). Importantly, quantification of HMGB1 release by ELISA revealed that HMGB1 release precedes ES-Cu–induced cell death in Calu1 cells (Figure 1B).

To further determine whether HMGB1 is a marker predicting cell sensitivity to cuproptosis, we examined the relationship between HMGB1 release and ES-Cu–induced cell death in MIA PaCa2 (a human pancreatic cancer cell line) cells and mouse embryonic fibroblasts (MEFs). As in Calu1 cells, HMGB1 release preceded ES-Cu–induced cell death in MEFs (Figures 1C,D). In contrast, MIA PaCa2 cells were relatively resistant to ES-Cu–induced cell death and lacked substantial HMGB1 release (Figures 1E,F).

To determine whether this cell death process is dependent on the induction of cuproptosis, we treated cells with cell death inhibitors. Among the various cell death inhibitors tested, including Z-VAD-FMK, necrostatin-1, and ferrostatin-1, only tetrathiomolybdate inhibited ES-Cu–induced cell death and HMGB1 release (Figures 1G,H). Despite ES-Cu–induced production of mitochondrial reactive oxygen species (ROS; Figure 1I), the selective mitochondrial ROS scavenger MitoQ10 failed to inhibit ES-Cu–induced cell death and HMGB1 release (Figures 1G,H). These findings demonstrate that the release of HMGB1 in cuproptosis is copper-dependent, rather than mitochondrial ROS-dependent.

To determine whether HMGB1 is a sensitive marker of apoptosis, we also examined the time course of the release of lactate dehydrogenase (LDH), another DAMP used to monitor cellular damage. Compared with HMGB1, LDH was released no earlier than 6 h after ES-Cu treatment (Figure 1J).

### AMPK inhibits HMGB1 release during cuproptosis

**FIGURE 2:**
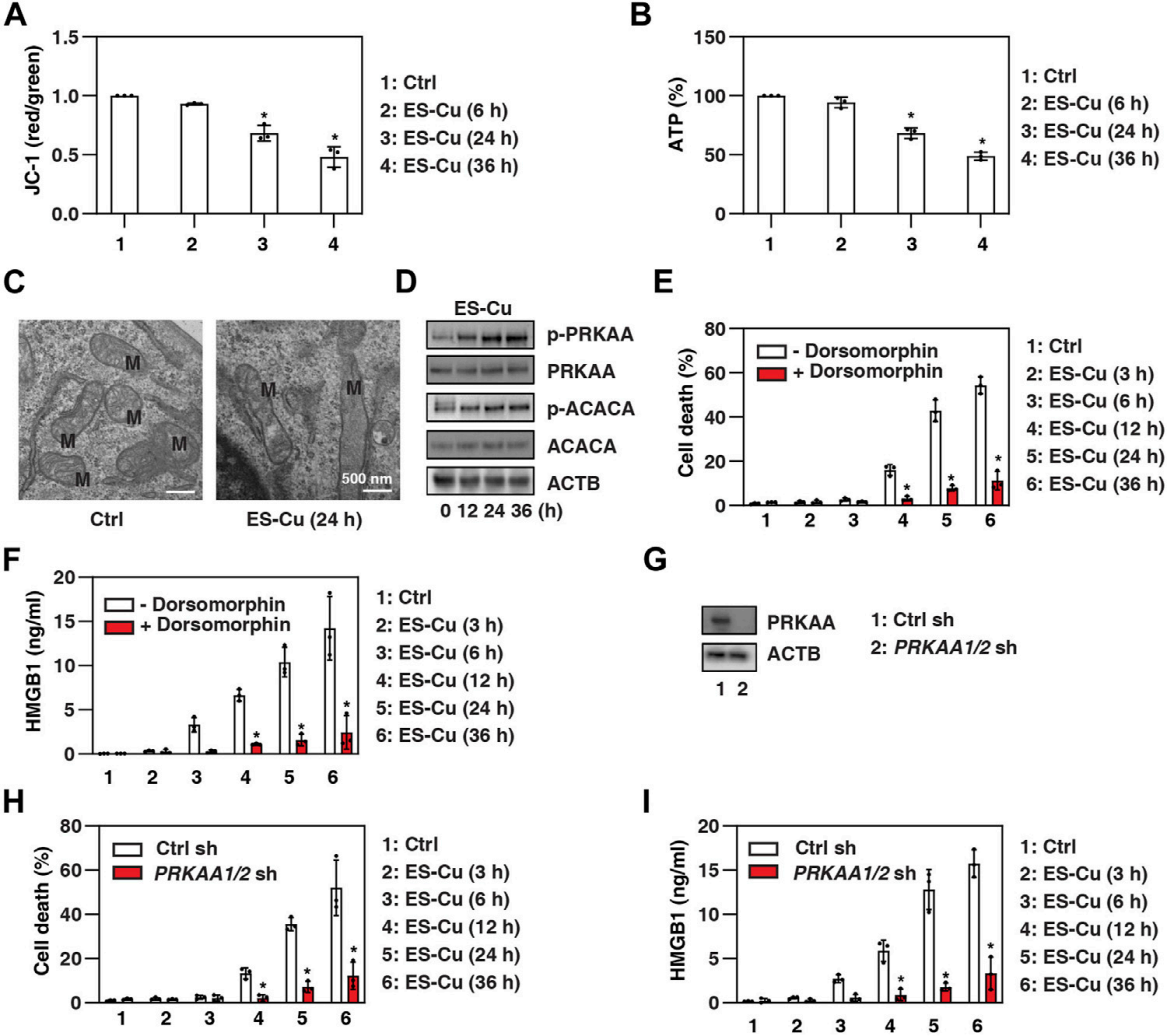
AMPK inhibits HMGB1 release during cuproptosis. (A–D) Analysis of mitochondrial membrane potential (JC-1 staining), intracellular ATP, mitochondrial ultrastructure (M = mitochondria), and protein expressions in Calu1 cells after treatment with ES-Cu (30 nM/30 nM) for the indicated times. (E,F) Calu1 cells were treated with ES-Cu (30 nM/30 nM) in the absence or presence of dorsomorphin (2 µM) for 3–36 h and then cell death and HMGB1 release were assayed (n = 3; *p < 0.05 versus control group, one-way ANOVA). (G) Western blot analysis of PRKAA expression in *PRKAA1/2*-knockdown Calu1 cells. (H,I) Wild-type and *PRKAA1/2*-knockdown Calu1 cells were treated with ES-Cu (30 nM/30 nM) for 3–36 h and then cell death and HMGB1 release were assayed (*n* = 3; *p < 0.05 versus control group, one-way ANOVA). The results in (A–I) are representative of those from three independent experiments.

Since mitochondrial dysfunction is the hallmark of cuproptosis (Tang et al., 2022), we analyzed the effect of ES-Cu on mitochondrial membrane potential using JC-1, a dye that can selectively enter mitochondria and reversibly change from green to red as membrane potential increases (Perelman et al., 2012). This assay showed that mitochondrial membrane potential was decreased by ES-Cu (Figure 2A). Contrary to a previous report (Tsvetkov et al., 2022), ES-Cu also caused adenosine triphosphate (ATP) depletion, a characteristic event of mitochondrial dysfunction (Figure 2B). Transmission electron microscopy confirmed that there was mitochondrial morphological damage, including a reduction or disappearance of mitochondrial cristae (Figure 2C). Since AMP-activated protein kinase (AMPK) acts as an intracellular energy sensor and its activity is regulated by metabolic signals (including relative levels of ATP) (Herzig and Shaw, 2018), we analyzed the effect of ES-Cu on protein kinase AMP-activated catalytic subunit alpha (PRKAA, best known as AMPKα), which is the main catalytic domain of the AMPK complex. Western blotting showed that PRKAA phosphorylation was upregulated by ES-Cu, whereas the total expression of PRKAA was not altered (Figure 2D). The phosphorylation of acetyl-CoA carboxylase alpha [ACACA, a bona fide substrate of AMPK (Herzig and Shaw, 2018)] was also upregulated by ES-Cu (Figure 2D), further supporting that the induction of cuproptosis is associated with AMPK activation.

Next, we investigated the impact of AMPK activation on cuproptosis and HMGB1 release. We first used the classical AMPK inhibitor dorsomorphin (also known as compound C) in Calu1 cells and MEFs. Dorsomorphin inhibited ES-Cu–induced cell death and HMGB1 release compared to vehicle controls (Figures 2E,F). Furthermore, the double knockdown of PRKAA1 (AMPKα1) and PRKAA2 (AMPKα2) by specific shRNAs also inhibited ES-Cu–induced cell death and HMGB1 release in Calu1 cells (Figures 2G–I). Thus, the activation of AMPK contributes to cuproptosis and HMGB1 release.

### HMGB1 is phosphorylated by AMPK during cuproptosis

**FIGURE 3:**
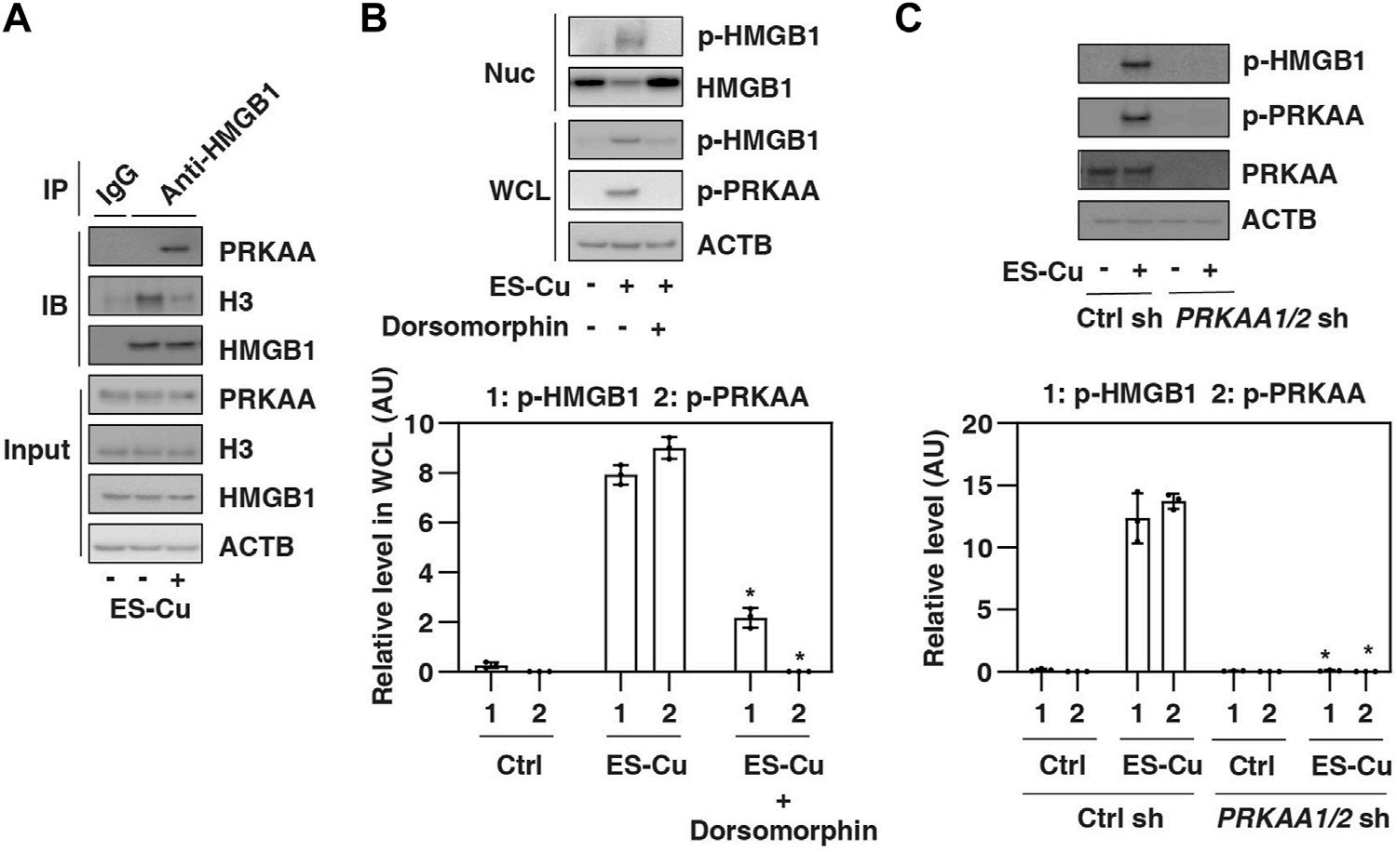
HMGB1 is phosphorylated by AMPK during cuproptosis. (A) IP analysis of the binding between HMGB1, PRKAA, and H3 in Calu1 cells following treatment with ES-Cu (30 nM/30 nM) for 6 h (B,C) Effects of dorsomorphin or the knockdown of PRKAA on phosphorylation of HMGB1 and PRKAA in Calu1 cells following treatment with ES-Cu (30 nM/30 nM) for 6 h. Protein semi-quantification is shown in the down panel (*n* = 3; *p < 0.05 versus control group, two-way ANOVA). WCL: whole cell extraction. Nuc: nuclear extraction. The results in A-C are representative of those from three independent experiments.

HMGB1 is a nuclear protein that undergoes posttranslational modification (e.g., phosphorylation) in the nucleus to translocate to the cytoplasm, where it is ultimately released into the extracellular space (Kang et al., 2014b). We next examined whether HMGB1 is a direct substrate of AMPK, leading to HMGB1 phosphorylation and release. We focused on the early phase of ES-Cu treatment, as this phase has HMGB1 release but no significant cell death (Figure 1). Immunoprecipitation experiments revealed an increase in the interaction of PRKAA and HMGB1 in Calu1 cells after 6 h of treatment with ES-Cu (Figure 3A). In contrast, the interaction between HMGB1 and histone H3 was decreased by ES-Cu (Figure 3A). These findings suggest a competitive relationship between PRKAA and H3 for binding to HMGB1 during cuproptosis.

Next, we determined whether AMPK regulates HMGB1 phosphorylation. Immunoprecipitation experiments showed that phosphorylated HMGB1 was increased in whole cell lysate and nuclear extraction by ES-Cu, a process blocked by the AMPK inhibitor dorsomorphin (Figure 3B). Similarly, the knockdown of PRKAA1/2 also inhibited ES-Cu–induced phosphorylation of HMGB1 in Calu1 cells (Figure 3C). Together, these findings suggest that HMGB1 is phosphorylated by AMPK during cuproptosis, which may contribute to the release of HMGB1.

### HMGB1 triggers AGER-dependent inflammation caused by cuproptotic cells

**FIGURE 4:**
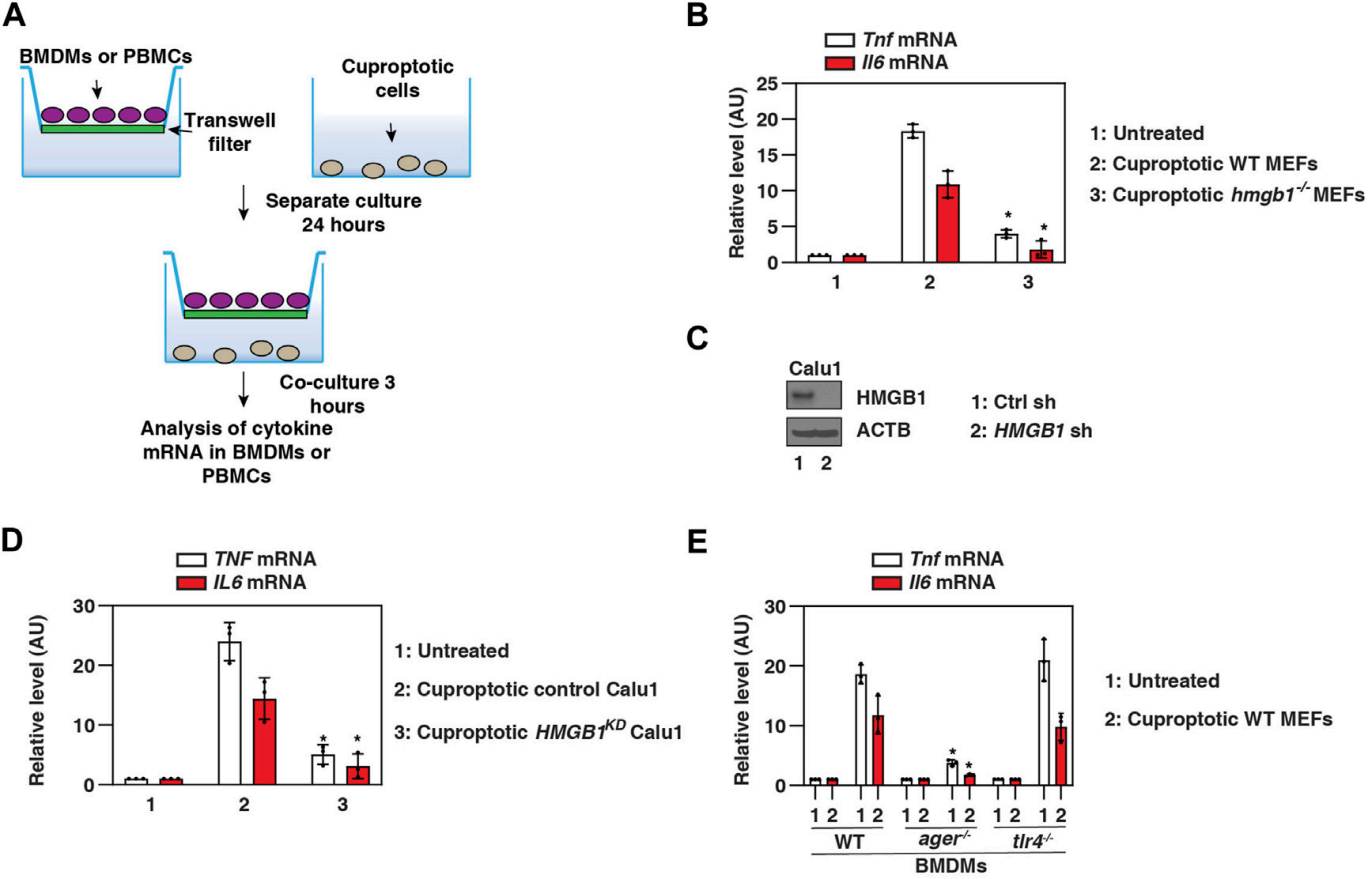
HMGB1 triggers AGER-dependent inflammation caused by cuproptotic cells. (A) Scheme of transwell systems for co-cultures of immune cells and cuproptotic cells. (B–D) Our qPCR assays show that TNF and IL6 mRNA levels in BMDMs or PBMCs were reduced in response to cuproptotic *hmgb1* ^−/−^ MEFs or HMGB1-knockdown Calu1 cells (*n* = 3; *p < 0.05 versus control group, two-way ANOVA). (E) A qPCR assay shows that cuproptotic wild-type MEF-induced upregulation of *Tnf* and *Il6* mRNA was inhibited in *ager* ^*−/−*^ BMDMs, but not in *tlr4* ^*−/−*^ BMDMs (*n* = 3; *p < 0.05 versus WT group, two-way ANOVA). The results in (B,D,E) are representative of those from three independent experiments.

To determine whether cuproptosis-related HMGB1 release directly triggers the inflammatory response, we challenged primary WT mouse bone marrow-derived macrophages (BMDMs) with ES-Cu–treated *hmgb1* ^*−/−*^ or WT MEFs (Figure 4A). Cuproptotic WT cells triggered the production of pro-inflammatory cytokines (e.g., tumor necrosis factor [TNF] and interleukin 6 [IL6]) more efficiently than cuproptotic *hmgb1* ^*−/−*^ cells (Figure 4B). HMGB1-knockdown cuproptotic Calu1 cells (Figure 4C) were also ineffective in activating primary human peripheral blood mononuclear cells (PBMCs) to produce TNF and IL6 (Figure 4D).

Several pattern recognition receptors are associated with extracellular HMGB1 activity. We next focused on TLR4 and AGER, the two major receptors for HMGB1 activity in immune cells (Andersson and Tracey, 2011; Kang et al., 2014b). Cuproptotic cell-induced TNF and IL6 production were impaired in *ager* ^*−/−*^ BMDMs compared with WT and *tlr4* ^*−/−*^ BMDMs (Figure 4E). These assays support the hypothesis that HMGB1 released from cuproptotic cells induces AGER-dependent inflammatory cytokine production in macrophages.

## Discussion

Heavy metal poisoning is due to the accumulation of certain metals in the body, resulting in cell death, impaired tissue function, and symptoms of inflammation (Tchounwou et al., 2012). Unlike iron-induced oxidative ferroptosis (Stockwell et al., 2017), the induction of cuproptosis is dependent on copper-induced mitochondrial stress (Tang et al., 2022). In this study, we provide strong evidence that AMPK participates in cuproptosis and HMGB1 release, leading to proinflammatory cytokine production. Our findings strengthen the DAMP hypothesis for human disease caused by pathological cell death (Huang et al., 2015).

Copper is a trace element that can switch through different redox states, influence the activity of many cellular enzymes, and sustain cellular homeostasis (Kardos et al., 2018). However, excessive copper overload can lead to cell death, including apoptosis (Rhee et al., 2013), and more recently, cuproptosis (Tsvetkov et al., 2022). Although mitochondria are the central hub for various cell death signals, cuproptosis and apoptosis use distinct mitochondrial stress signaling pathways to trigger death. Consistent with previous studies (Li and Yuan, 2008; Tsvetkov et al., 2022), we demonstrated that caspase activation required for apoptosis is not essential for cuproptosis, although in both forms of cell death, dying cells have a decline in mitochondrial membrane potential.

During the induction of cuproptosis, the aggregation of impaired mitochondria results in less ATP synthesis and more ROS production (Bock and Tait, 2020). Although mitochondrial ROS is a signaling molecule for triggering apoptosis, this pathway is not critical for cuproptosis (Tsvetkov et al., 2022). In the current study, we demonstrated that ATP depletion is responsible for AMPK activation and subsequent cuproptosis. Our findings support that cuproptosis is a type of metabolic death involving not only impaired protein degradation pathways, but also abnormal energy stress (Tang et al., 2022). Calcium has also been found to activate AMPK through the calmodulin-calcium/calmodulin–dependent protein kinase 2 (CAMKK2) pathway (Woods et al., 2005), whether or not mitochondrial calcium uniporters are involved in the regulation of cuproptosis remains to be investigated (Zhao et al., 2019). Nevertheless, the AMPK-mediated autophagy pathway may affect the aggregation or degradation of toxic mitochondrial proteins, resulting in feedback mechanisms to control cuproptosis sensitivity (Herzig and Shaw, 2018).

Our findings highlight that HMGB1 is a novel phosphorylated substrate of AMPK that contributes to the release of HMGB1. Under normal conditions, HMGB1 is tightly bound to components of the nucleosome, namely histones and DNA (Lotze and Tracey, 2005). During cellular stress, HMGB1 is translocated from the nucleus to the cytoplasm and then released (Wang et al., 1999; Scaffidi et al., 2002). This process is regulated by many factors, including posttranslational modifications of HMGB1 (Kang et al., 2014b). Lipopolysaccharide-induced secretion of HMGB1 from macrophages requires protein kinase C-mediated phosphorylation of HMGB1 (Oh et al., 2009). Our current study shows that AMPK is required for HMGB1 phosphorylation in lung cancer cells during cuproptosis, leading to the dissociation of HMGB1 from histones. These findings provide a mechanism to explain the active process of HMGB1 release in the early stages of cuproptosis, although the late release of HMGB1 is passively due to plasma membrane rupture.

We revealed that HMGB1 is a mediator of cuproptotic cell-induced macrophage activation. Macrophages are professional phagocytes closely related to wound healing, involved in everything from suppressing inflammation to clearing cellular debris and coordinating tissue repair (Lemke, 2019). Once this process is disrupted, dead or dying cells activate inflammation, causing massive tissue damage (Lemke, 2019). Our study confirms that AGER, but not TLR4, is required for the inflammatory activity of HMGB1 during cuproptosis. As a stress-related highly abundant protein, HMGB1 release is a universal marker of various types of cell death (Scaffidi et al., 2002; Lamkanfi et al., 2010; Wen et al., 2019; Fang et al., 2021). The combined use of other DAMPs is important to differentiate between different cell death-induced immune responses (Zindel and Kubes, 2020).

In summary, we report a novel metabolic mechanism of cuproptosis and establish that HMGB1 is a substrate of AMPK, leading to inflammation. Targeting the release and activity of HMGB1 may prevent cuproptosis-related inflammatory diseases.

## Data Availability

The original contributions presented in the study are included in the article/Supplementary Material, further inquiries can be directed to the corresponding authors.

## Abbreviations

ACACA, acetyl-CoA carboxylase alpha; AGER, advanced glycosylation end product-specific receptor; AMPK, AMP-activated protein kinase; ATP, adenosine triphosphate; BMDM, mouse bone marrow-derived macrophage; DAMP, damage-associated molecular pattern; HMGB1, high-mobility group box 1; IL6, interleukin 6; MEF, mouse embryonic fibroblast; PBMC, peripheral blood mononuclear cell; PRKAA, protein kinase AMP-activated catalytic subunit alpha; ROS, reactive oxygen species; TLR4, toll-like receptor 4; TNF, tumor necrosis factor.
