# Supplementary figures and images for: HMGB1 is a mediator of cuproptosis-related sterile inflammation
Source: Front Cell Dev Biol. 2022 Sep 21;10:996307. doi: 10.3389/fcell.2022.996307 (PMC9534480; doi:10.3389/fcell.2022.996307)

FIG.2D

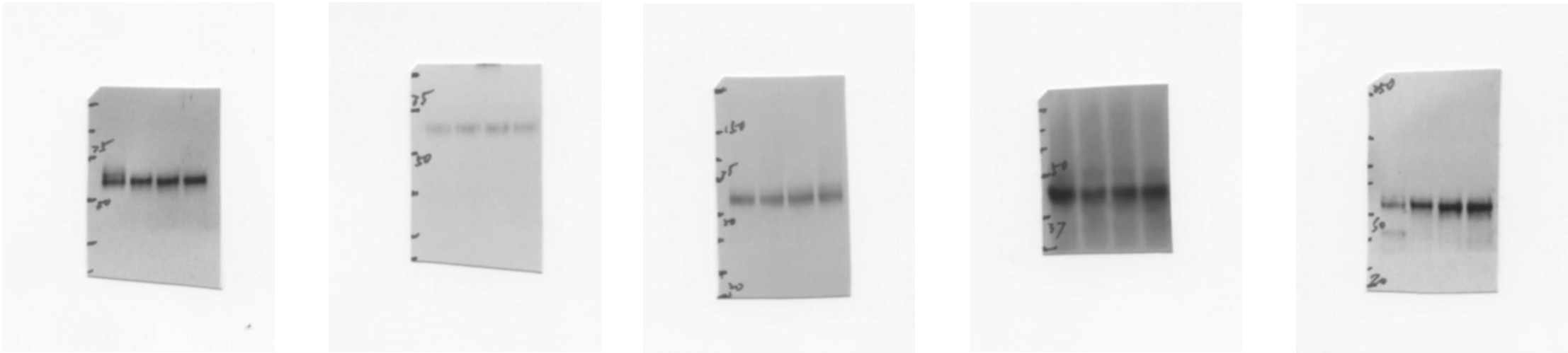

FIG.2G

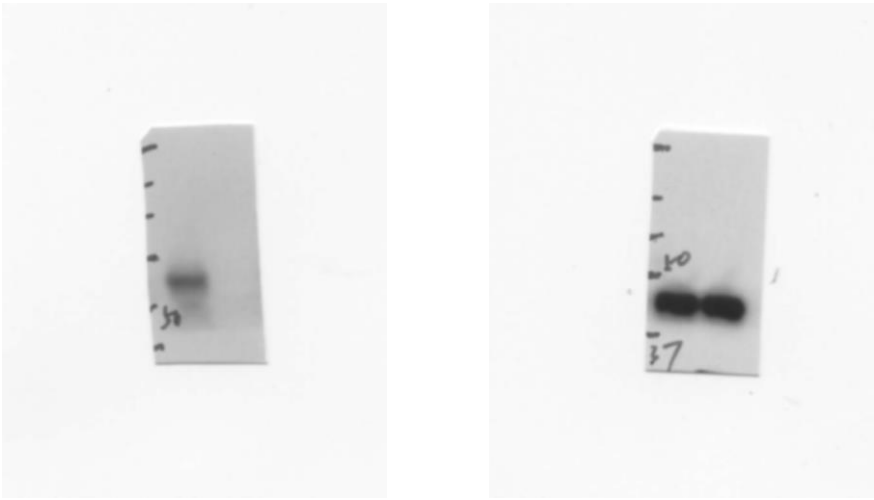

FIG.3A

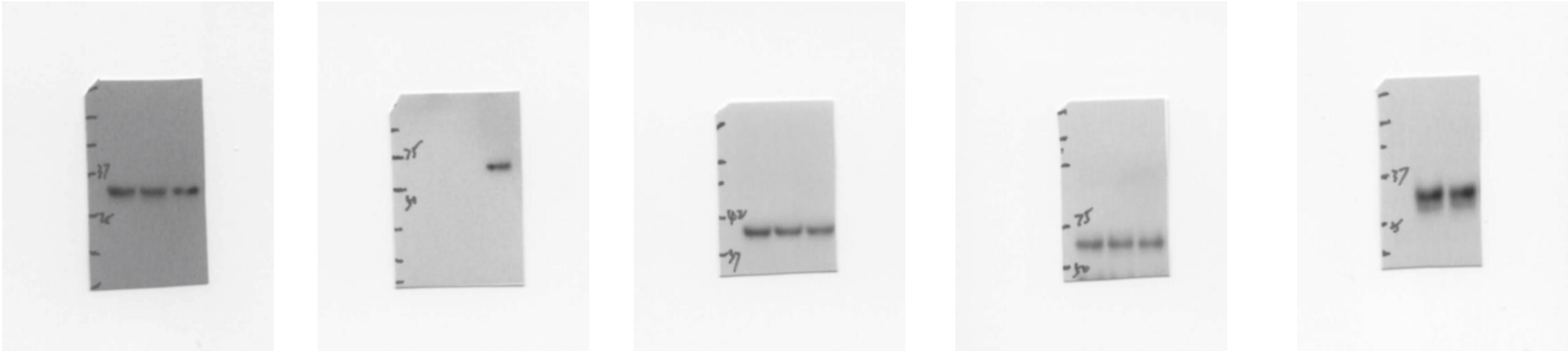

FIG.3B

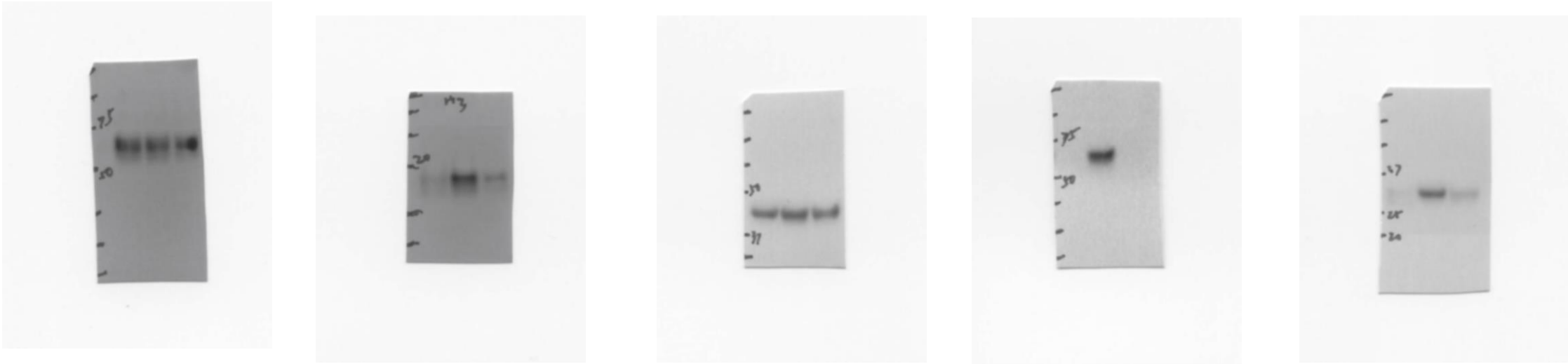

FIG.3C

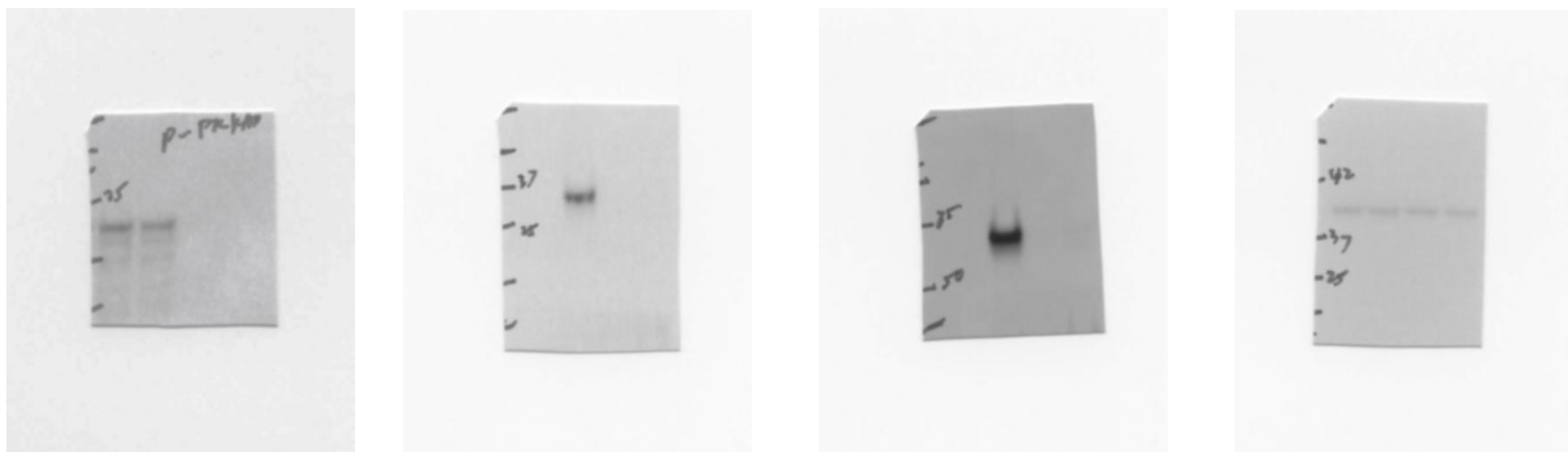

FIG.4C

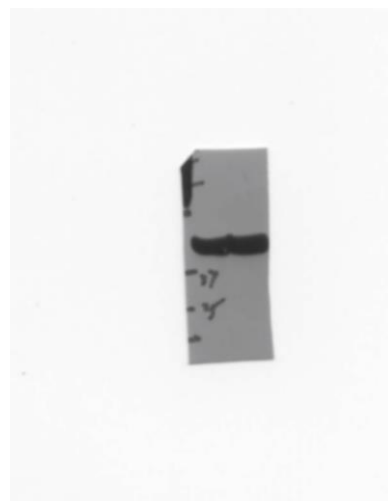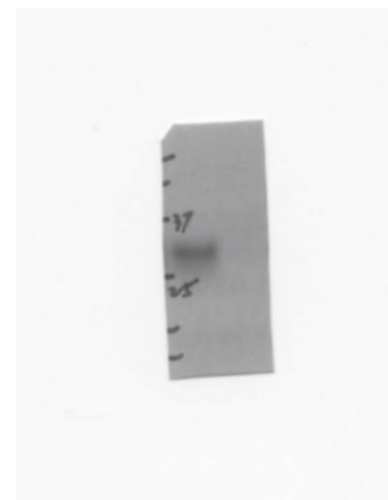

FIG.2C

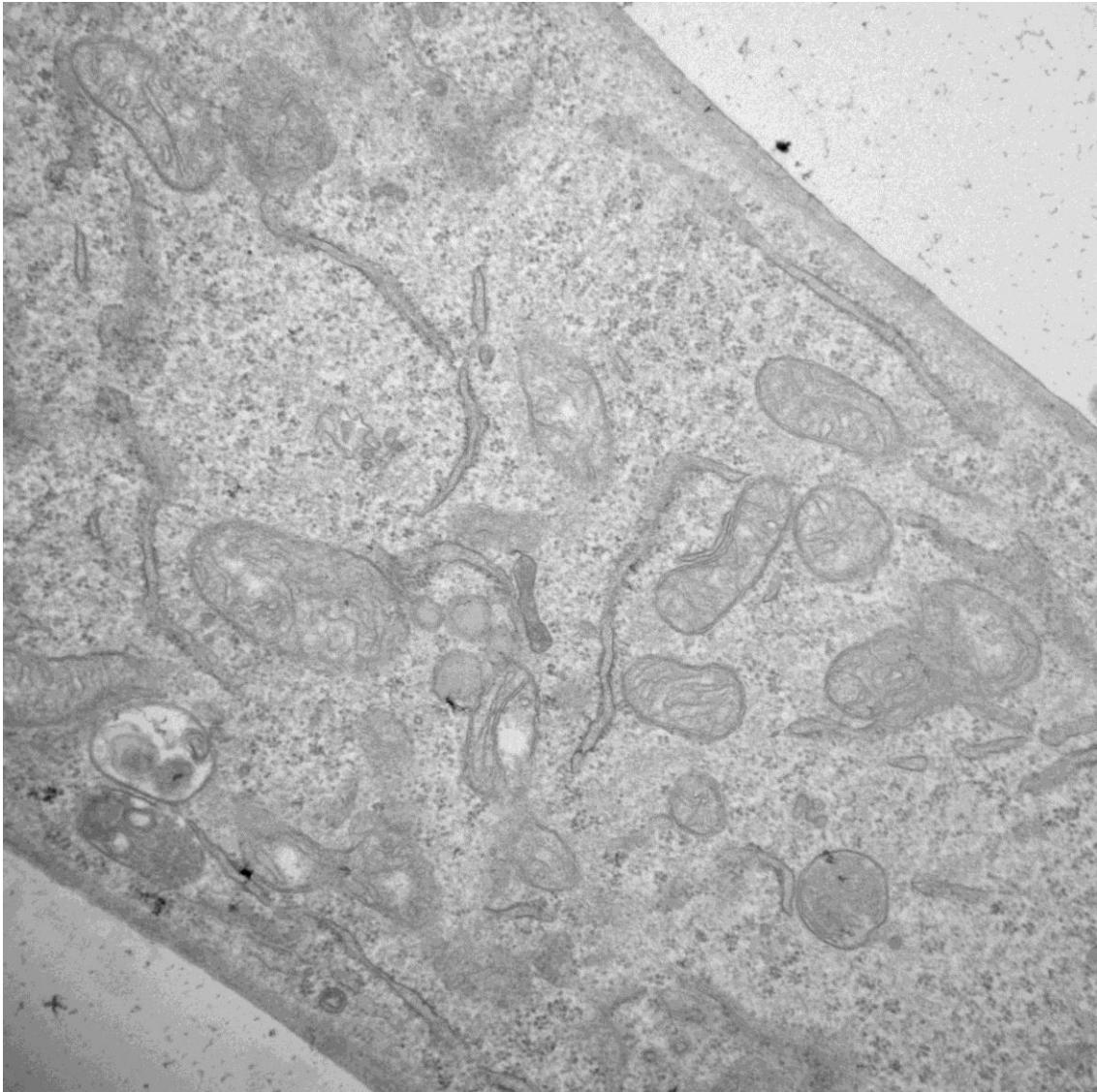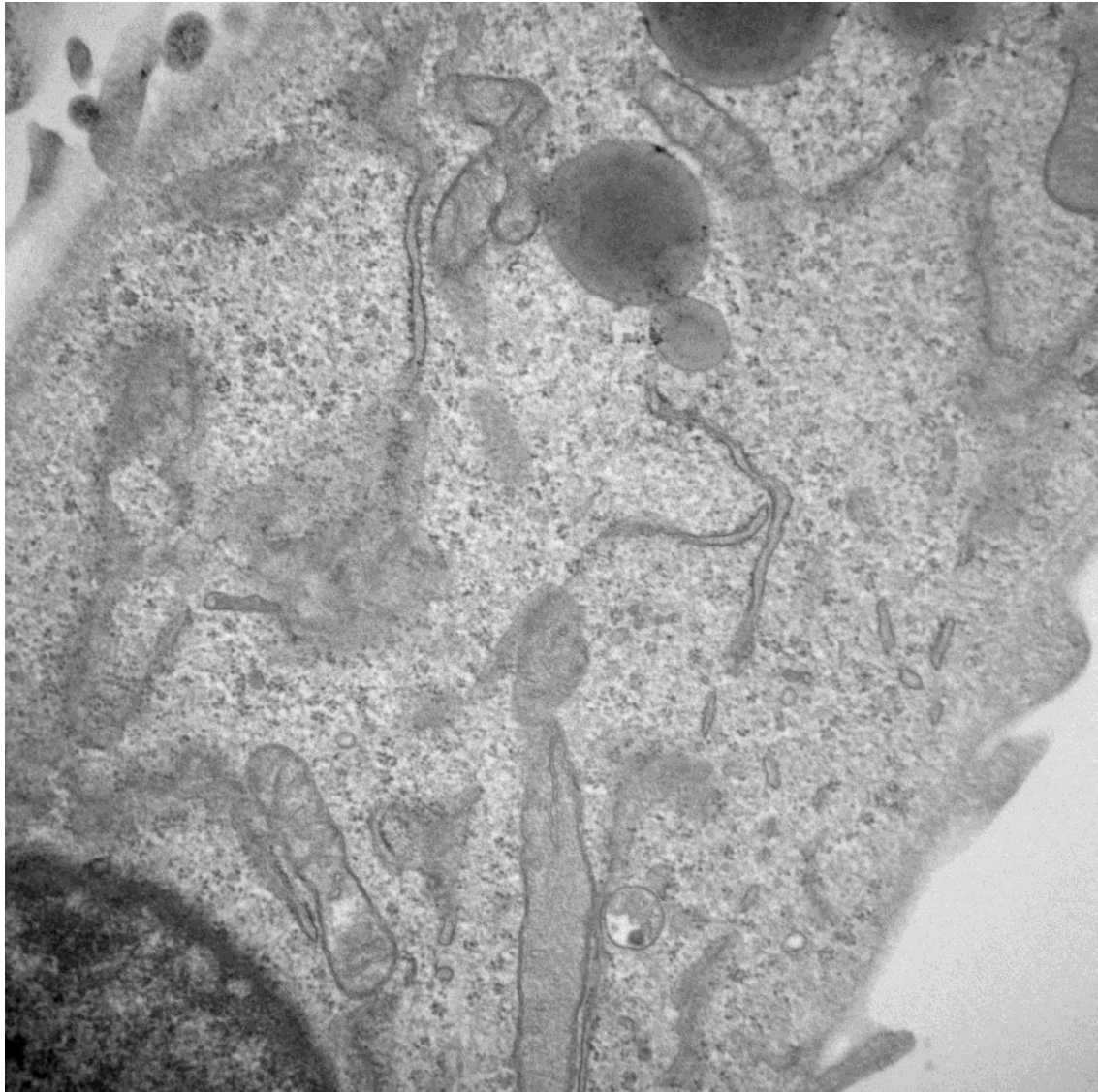

Supplement: Supplementary file 2 [file DataSheet1.PDF]
